# EasyMap - An Interactive Web Tool for Evaluating and Comparing Associations of Clinical Variables and Microbiome Composition

**DOI:** 10.3389/fcimb.2022.854164

**Published:** 2022-05-13

**Authors:** Ehud Dahan, Victoria M. Martin, Moran Yassour

**Affiliations:** ^1^ Microbiology and Molecular Genetics, Faculty of Medicine, The Hebrew University of Jerusalem, Jerusalem, Israel; ^2^ Department of Pediatrics, Massachusetts General Hospital, Boston, MA, United States; ^3^ School of Computer Science & Engineering, The Hebrew University of Jerusalem, Jerusalem, Israel

**Keywords:** microbiome, multivariate linear regression, clinical association, interactive, webtool

## Abstract

One of the most common tasks in microbiome studies is comparing microbial profiles across various groups of people (e.g., sick vs. healthy). Routinely, researchers use multivariate linear regression models to address these challenges, such as linear regression packages, MaAsLin2, LEfSe, etc. In many cases, it is unclear which metadata variables should be included in the linear model, as many human-associated variables are correlated with one another. Thus, multiple models are often tested, each including a different set of variables, however the challenge of selecting the metadata variables in the final model remains. Here, we present EasyMap, an interactive online tool allowing for (1) running multiple multivariate linear regression models, on the same features and metadata; (2) visualizing the associations between microbial features and clinical metadata found in each model; and (3) comparing across the various models to identify the critical metadata variables and select the optimal model. EasyMap provides a side-by-side visualization of association results across the various models, each with additional metadata variables, enabling us to evaluate the impact of each metadata variable on the associated feature. EasyMap’s interface enables filtering associations by significance, focusing on specific microbes and finding the robust associations that are found across multiple models. While EasyMap was designed to analyze microbiome data, it can handle any other tabular data with numeric features and metadata variables. EasyMap takes the common task of multivariate linear regression to the next level, with an intuitive and simple user interface, allowing for wide comparisons of multiple models to identify the robust microbial feature associations. EasyMap is available at http://yassour.rcs.huji.ac.il/easymap.

## Introduction

Examining microbiome differences in the context of clinical changes has become a widely-popular task in many academic and industry contexts (Belkaid and Hand, 2014; Borbet et al., 2019; Niu et al., 2021; Sorbara and Pamer, 2022). The ease of collecting stool samples (compared to blood or biopsy samples), together with the growing evidence of the microbiome’s contribution to human health (Becattini et al., 2016), makes the gut-microbiome case/control cohort design even more commonly used in the field (Zhu et al., 2013; Duvallet et al., 2017; Huang et al., 2021).

While animal-studies are conducted in a well controlled environment, they often do not represent human health in sufficient accuracy (Nguyen et al., 2015; Nagpal et al., 2018; Ma, 2021). On the other hand, in human cohorts we have the great challenge of dealing with all the additional characteristics that vary in the human population, such as age, diet, lifestyle, which are known confounders of the gut microbiome, and can bias our results (Vujkovic-Cvijin et al., 2020; “De-Confounding Microbiome Association Studies” n.d.; Devkota, 2016; Bartolomaeus et al., 2020). In an attempt to address this inherent bias in human studies, the field always strives to establish as large cohorts as possible, such as the UK biobank, LifeLines, and the TEDDY cohort (“UK Biobank - UK Biobank” n.d.; TEDDY Study Group, 2008; Davidson-Pilon, 2019). However, it is very difficult and costly to establish and manage large cohorts, and not all clinical manifestations enable such large cohorts, and even in these large numbers, computational methods that take into account the confounding factors are much in need (Jessica and Hanson, 2020).

An additional challenge in microbiome case/control studies is the interoperability of the results. While some machine-learning algorithms perform well on large datasets (Chen et al., 2020; Gou et al., 2021; Carrieri et al., 2021), they are often discriminative in the case/control task without revealing additional information on the underlying reason for the success of their method. Alternatively, the results will highlight specific microbial features that may play a role in the examined clinical manifestation (Oh and Zhang, 2021), which can be further studied from a medical- or a basic-science perspective, as the basis for further studies understanding the mechanisms underlying this association (Aasmets et al., 2021).

A common approach in all case/control studies is the use of multivariate linear regression models that take into account the variables of interest (i.e., microbiome composition) together while accounting for the confounding variables mentioned above (Ramette, 2007; Xia and Sun, 2017; Bodein et al., 2019; Raimondi et al., 2021). There are many packages (R, python, and independent tools) that perform this task, and one of the most-popular tools in the context of microbiome studies is MaAsLin2 (Mallick et al., 2021; S. Ma et al., n.d.; Ma et al., 2021; Zhang et al., 2021). It is especially useful in microbiome studies due to the data transformation (arcsine square-root transformation, Methods), outlier removal, presentation of results and its overall ease of use. Oftentimes, researchers will run multiple models in an attempt to find the ideal model that explains the data best, without overfitting. However, the routine task of comparing MaAsLin results across multiple models is challenging. First in running the multiple models, but more importantly in interpreting the subtle differences in their results.

Here, we present EasyMap, a user-friendly interactive web-based tool that enables running multiple linear regression models and comparing across their results in a graphical manner. While EasyMap was designed to analyze microbiome data, it can handle any other tabular data with numeric features and metadata variables. EasyMap enables the users to upload their own data, construct multiple models, and run the analyses using MaAsLin2, regardless of their computational background and expertise. EasyMap also improves the usability of viewing the significant results, in an interactive high- and low-level visualization of the results. Most importantly, EasyMap provides an easy framework for comparing across models, stratifying the linear regression results by additional variables, and eventually assisting in choosing the optimal model for the data.

## Methods and Implementation

### Tool Implementation

The EasyMap web tool was developed using the *shiny* R package (version 1.6.0), and it uses the *MaAsLin2* R package (version 1.4.0) for multivariate linear regression. All code is available for download on the Yassour lab git repository (Dahan, 2021). EasyMap is available for public use at https://yassour.rcs.huji.ac.il/easymap.

### Multivariate Linear Model

EasyMap provides an easy web-based system to perform a multivariable association analysis between microbial features changes to clinical measurement (metadata). Analysis modules include preprocessing, normalisation and transformation and produce a statistically significant output including correction for multiple tests (see below). All user choices are processed to MaAsLin2 format, and are run with the default MaAsLin2 parameters.

**Figure 1:**
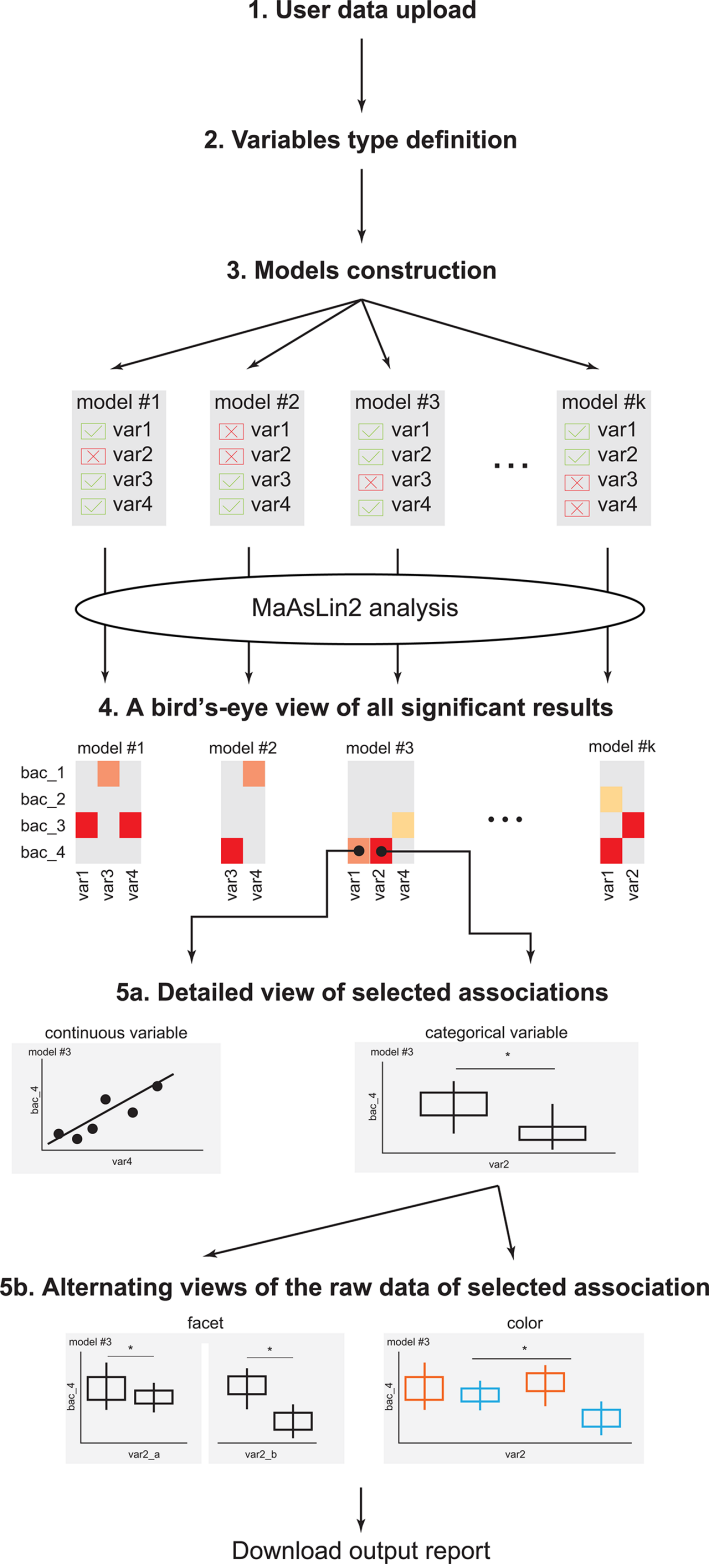
EasyMap workflow. A schematic representation of the EasyMap workflow. Step 1 - User data upload: Enable users to upload their separator based files (comma, tab, etc.). The case study data can be loaded using the ‘example’ button. Step 2 - Variables type definition: Define the variables in the data as categorical or continuous, and as random or fixed effects for the linear model. Step 3 - Model construction: Include or exclude variables for each model, and select the reference value for each variable. Step 4 - A birds-eye view of all significant results: An interactive heatmap presenting all significant results (calculated by running MaAsLin2 on the constructed models). The results from the various models are displayed side-by-side for optimal comparisons, and the display parameters (such as the specific microbial features, models, and significance threshold) are shown on the left. Step 5a - Detailed view of selected associations: Once the user clicks on a specific association in the heatmap, a detailed view of that result is displayed below. Associations with categorical or continuous variables are displayed as box plots, or scatter plots, respectively. Plots include q-values extracted from MaAsLin2, with brackets indicating the specific comparisons. Step 5b - Alternating views of the raw data of selected association: The detailed plots can be further stratified in two options: (a) coloring by a specific variable (keeping the x-axis as before); and (b) faceting by a specific variable, which splits the x-axis based on the values of the selected variable. Download output report: The heatmap and the presented detailed plots can be downloaded as a pdf file. * = statistical value (p or q value) is less than statistic threshold.

Variables in the model are defined as either random- or fixed-effect, based on the user definition (**Figure 1**, Step 2). Briefly, fixed variables impact all samples equally, while random variables impact samples differentially, based on the value of the random-effect variable (Srinivasjois, 2021; Kanters, 2022).

### Preprocessing and Normalization

After loading the input data, total sum scaling normalization is applied to each sample, and then microbial features with a total normalized sum of less than 0.0001 were removed. Next, abundance data were transformed with the arcsine square root-transformation (AST) (Biometry: The Principles and Practice of Statistics in Biological Research, 1981). Microbiome data is often sparse and zero-inflated, thus the arcsine square root transformation is an ideal choice to spread the abundance values, but maintain zero abundance. By default, MaAsLin2 uses the na.exclude function, which excludes the Not available (NA) values from the multivariate linear regression calculations, but keeps these values for the association visualization (will appear as a separate category, without any statistical calculation). Specifically, if the model includes the variable which has an NA value for some samples, these will not be included in the statistical analysis, but will be presented in the visualization.

### Statistical Analysis

Nominal p-values across all associations in the resulting heatmap were adjusted using Benjamini-Hochberg FDR method performed by MaAsLin2, and the coefficient and resulting q-values appear in the box plots with the corresponding brackets. After ‘faceting’ the box plot, the tool presents the p-values calculated by a two-sided t-test (using the *ggpubr* R package; version 0.4.0), based on all the samples that appear in the plot (without any MaAsLin2 filtering).

### Box Plots

In the box plots each dot represents one sample, the middle line represents the median of the distribution, and the box boundaries represent the first and third quartiles. The y-axis represents the transformed relative abundance of a microbial feature bacteria (AST, Methods) and the x-axis is the selected effect variable. All box plots were generated by the ggplot2 R package (version 3.3.3). See statistical analysis Methods section above for q-values and p-values description.

### Case Study Data

From the GMAP prospective observational healthy infant cohort, selected infants diagnosed with food protein-induced allergic proctocolitis (FPIAP) who had a minimum of 4 longitudinal stool samples and selected matched controls for each who met the same sampling criteria. 16S rRNA gene libraries targeting the V4 region of the 16S rRNA gene were sequenced on an Illumina MiSeq 300 (raw sequencing data can be found on NCBI BioProject PRJNA730851). Total of 954 samples remain for further analysis.

### Running EasyMap on Other, Non-Microbiome, Data

We developed EasyMap to assist us in analyzing microbiome data. However, it can also be used in additional contexts where multivariate linear models are commonly used, maintaining all its added value. The input data format is described below (see Step 1: Input data upload), yet specific attention should be paid to the AST transformation that is applied automatically on the feature data input, which is not optimal for all datasets. The user can choose to not apply this transformation on the uploaded data.

## Workflow of EasyMap + Case Study Example

### Cohort and Data Description

Here, we describe the step-by-step flow of the EasyMap interactive tool, available at http://yassour.rcs.huji.ac.il/easymap (also presented in **Figure 1**). To demonstrate the performance of EasyMap and make it easier to use, we carried out a case study (Martin et al., 2021) and added a short explanation at the end of each step. This case study examines the early childhood microbiome in allergic infants. In this project, 160 infants were longitudinally sampled during the first year of life (6 time points). During this period, 81 infants were diagnosed with food-protein induced allergic proctocolitis (FPIAP), specifically to cow’s milk proteins. To characterize the microbial profiles, 16S sequencing was performed on all infant stool samples (N=954). Here, we demonstrate the advantage of using the EasyMap web tool to investigate statistical significant associations between microbial features and clinical data (i.e., allergic diagnosis), using multivariate linear regression models.

### Step 1: Input Data Upload

The first step is uploading the user input data, which is a separator based file (csv, tsv etc.) including all relevant data: clinical metadata variables and taxonomic features’ abundance for each sample (**Figure 1**). This file should follow MaAsLin (Morgan et al., 2012) format (largely described below) and should have a header line. Suppose there are n metadata variables and m taxonomic features, the input file will have three sections of columns: (a) The first column will contain the sample ID, which is a unique identifier of samples; (b) The next n columns will contain the metadata variables, where each of the variables can be either all strings or all numeric but not mixture. These can include clinical measurements and also other information, such as subject ID, or collecting clinic; (c) The last m columns will contain the abundance of the taxonomic features (relative or absolute). All abundance data will be normalized by total sum scaling (TSS) normalization (MaAsLin2 default) and then will be transformed by the arc-sinus transformation (AST, Methods). The user can choose not to apply the AST transformation on the uploaded data by unchecking the AST checkbox in Step 3 (model construction).

Users can upload their separator based files (comma, tab) through the ‘Upload Files’ tab. Once uploaded, all the identified columns will appear and the user can click the “Submit” button and continue with the analysis. If there is any problem with parsing the file, the error will be presented to the user.

### Step 1 - Case Study

**Figure 2:**
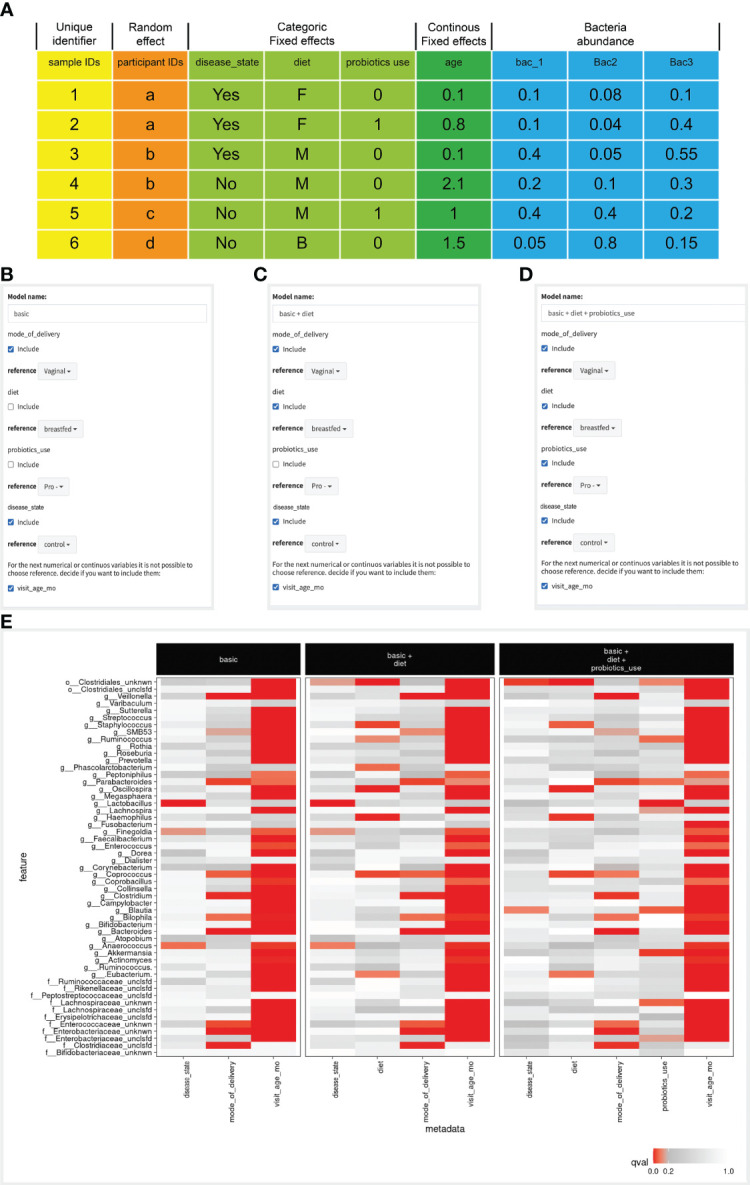
Defining the variables, constructing the models and comparing the results. **(A)** Input file example, where column colors represent different variable types (appear at the top of the table), and the first row is the table’s header. **(B–D)** Screenshots of the model construction step. Checkbox values next to each variable indicate if it is included in each model, and the reference value for each variable is selected in the dropbox. The last variable, named ‘visit_age_mo’ is defined as a continuous variable, therefore its reference is assigned as zero. **(E)** A heatmap showing the birds-eye view of all significant results across all models, from the MaAsLin2 analysis. Each box represents a model, rows are microbial features, and columns are model variables. Each entry in the heatmap is colored by the significance of the association of the specific microbial feature with the specific model variable.

In this case study, we considered six clinical variables that were collected in our cohort, and are also known to have an impact on gut microbiome composition: mode of delivery (vaginal or C-section), age (at time of visit), use of probiotics in the first year of life, infant diet at each time point (breastfed, formula-fed, mixed); and finally the disease status (case/control). In this study we are searching for microbial features that are associated with the disease status, taking into account all other clinical variables (top of this file is presented in **Figure 2A**). Clicking on the ‘example’ button loads a sample of the case study data.

### Step 2: Variables Type Definition

After uploading the data, it is necessary to define the type of all clinical metadata variables (**Figure 1**). First, the user selects the column that represents the unique sample ID. Second, the user selects the variables that will be used as *random effects* in the linear model (see Methods). The model will account for these variables, but will not search for associations between the random variables and the microbial features. Next, the user selects the fixed effect variables, which can be assigned as either continuous or categorical. All variables that remain unselected are automatically defined as the microbial features, thus all clinical variables must be selected as either random or fixed effect variables.

Categorical variables are automatically sorted alphabetically (for example, always, never, sometimes), however, if the user has a specific relevant order, the variable values can include a prefix to maintain this order (like, a_never, b_sometimes, c_always). Numeric variables that have four or less unique values will be treated as categorical variables. Once all variables are defined an “approve” button will appear at the bottom of the screen.

### Step 2 - Case Study

In this case study there were 954 samples from 160 different infants. We defined the infant ID as a random variable such that multiple samples from the same infants will be accounted for together, and not as independent measurements. Next, we defined delivery mode, disease status, probiotic use and infant diet as categorical variables. All variables other than diet have two values, and diet has three (breastfed, mixed or formula). Finally, we defined the age at the time of visit as a continuous variable, and all remaining variables are left as the microbial features (the relative abundance of each bacteria in each of the samples; **Figure 2A**).

### Step 3: Model Construction

When searching for statistical-significant associations, we first need to choose the clinical variables that our model should account for. These variables are usually chosen based on prior understanding of the clinical situation, and also including factors that are known to impact the microbial community composition. Naively, one can include all collected variables in the model, however, including too many variables would lead to overfitting the data, and diluting the signal across too many variables, potentially missing the significant association altogether. Oftentimes, we choose multiple models, each containing a different set of examined variables, with the aim to compare the results across these models. EasyMap was built to enable a comprehensive comparison across various models, thus highlighting the strong, consistent associations across multiple models.

After defining and approving the variables (as described in step 2) the user will next move to selecting the variables to be used in the first model (**Figure 1**). In the case of categorical variables, the user can also specify the reference value to be used for each variable. For example, if delivery_mode has two possible values: “C-section” or “vaginal”, the user can specify that “vaginal” will be the reference value. By default, the tool sorts the values alphabetically and the first value is used as reference. In the example above this would have been “C-section”.

Additional models can be added by clicking on the “add new set” button, and repeating this selection step for each model. By default, the new model is initiated with the selection of variables of the most recently defined model. While many models can be added and compared across, it impacts the total running time and the ease of results viewing in the next step, thus comparing 3-5 models seems ideal.

### Example Step 3 - Case Study

In this case study we wanted to find a microbial feature that is associated with disease status. We wanted to examine the contribution of a specific clinical variable to the associations we found. Here, we focused on the impact of infant diet and probiotics use on the microbial associations with disease status. Therefore we considered three models: (1) Including the case/control, mode of delivery and visit age variables as a base model. (2) model 1 variables + infant diet; and (3) model 2 variables + probiotic use (**Figures 2B–D**). We were interested to see whether associations found in model 1 remained when adding the diet and probiotic use variables, which will be revealed in the next step.

### Output Description

The output of the EasyMap is composed of two sections: A heatmap of all significant results and a detailed view of selected associations (for example using box plots), with the ability to facet and color the raw data. All the results that are shown on the screen (heatmap with the detailed plots) can be exported to a pdf file.

### Step 4: A Birds-Eye View of All Significant Results

The first step in comparing the models is a high-level comparison of all microbial associations that were found to be significant in at least one model. Heatmap color represents the significance, and by default, the FDR q-value threshold is set to be 0.2 (only associations that pass this threshold appear in color). The user can further filter the presented microbial features, using the drop-down menus on the left. The user can select a different threshold, and also choose which models to include in the heatmap (**Figure 1**).

### Example Step 4 - Case Study

**Figure 3:**
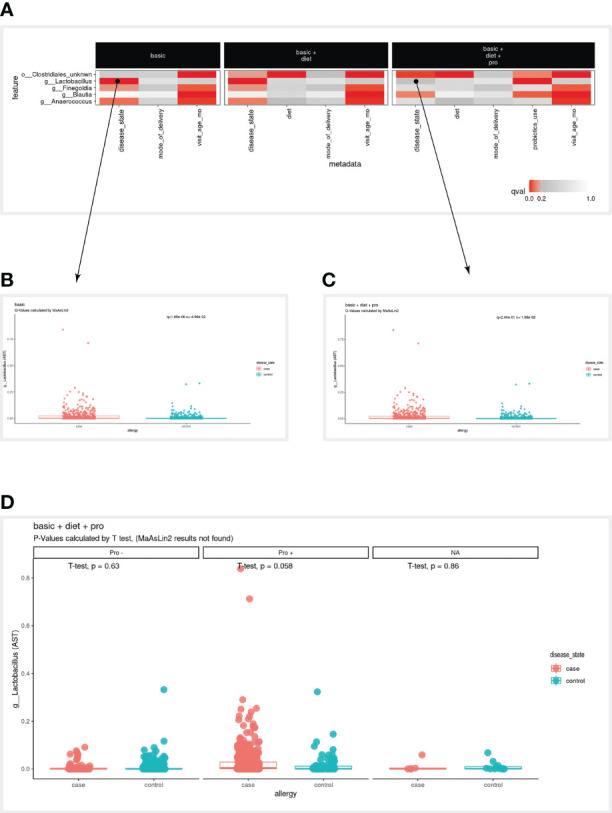
Case study results. **(A)** A subset of the full heatmap, showing the three tested models with only five selected microbial features (rows). **(B, C)** Clicking on the heatmap entries marked with a circle, generates these box plots, displaying the linear association of *g:Lactobacillus* with the disease variable, in the ‘basic’ model **(B)** and from ‘basic + diet + pro’ model **(C)**. In the box plots, each dot represents a sample, where the y-axis is the relative abundance of the microbial feature (AST, see Methods), and the x-axis is the values of the disease variable. **(D)** Box plot as in **(C)** stratified by values of the probiotics_use variable (Pro-, Pro+, NA). NA, Not available.

When examining the bird-eye view of significant results of our three models (**Figure 2E**), the first clear observation is that infant age was strongly correlated with most microbial features (**Figure 2B**). When examining the disease variables/column in the basic model, we found three significant associations (*g:Lactobacillus*, *g:Finegoldia* & *g:Anaerococcus*). However, in the two additional models (models 2 & 3), these associations are not significant anymore, and additional significant associations are detected (*o:Clostridiales_unknwn* in models 2 & 3, and *g:Blautia* in model 3). To enable a simpler comparison we have subset the heatmap to display only the microbial features mentioned above (**Figure 3A**). Interestingly, in the case of *g:Lactobacillus*, there was still a significant association in model 3, only with the probiotic use variable (**Figure 3A**). This shift indicated that once we added the probiotic use to the model, it better explains the different *g:Lactobacillus* abundance across the disease groups.

### Step 5a: Detailed View of Selected Associations

One unique and useful feature of EasyMap is the ability to toggle quickly between the bird’s eye view of all associations in the heatmap and zooming in on specific associations of interest (**Figure 1**). When the user hovers on a single cell in the heatmap, the cell is highlighted, and the relevant microbial feature together with the selected model, and associated clinical variable appear as text at the bottom of the panel. When the user clicks on a certain cell in the heatmap, the bottom panel is populated with a detailed plot showing the relative abundance (AST, if it was transformed, Methods) of the selected microbial feature by the selected clinical variable (this can be either a box plot for a categorical variable or a scatter plot for a continuous clinical variable). Note that if the relative abundance values (y-axis) are arc-sinus transformed thus can exceed 1, and range in [0, 1.57079]. The detailed plot also displays the q-values that are outputted by MaAsLin2 for all tested associations in this variable (using brackets comparing each value to the selected reference). Significance analysis appears for all possible comparisons between the reference and other values, with their respective q values, even for the non-significant comparisons.

### Step 5b: Alternating Views of the Raw Data of Selected Associations

Finally, to include additional metadata to the existing plot, the user can facet the box plot and/or color the dots, by a specific variable (**Figure 1**). When the plot is faceted, the MaAsLin2 q-values are removed from the plot, and instead a t-test is performed, and p-values are presented. Finally, the user can color the dots based on the categorical variables of the model, and add labels to the dots, based on the random variables of the model.

### Example Step 5 - Case Study

To further examine the case of *g:Lactobacillus*, we clicked on the square that corresponds to the *g:Lactobacillus* row in the disease column on model 1, which displayed the box plot on the bottom panel (**Figure 3B**). The MaAsLin2 q-value was 1.65e-06, indicating that *g:Lactobacillus* is highly correlated with disease status. However, when clicking on the square that corresponds to the *g:Lactobacillus* row in the disease column on *model 3* (**Figure 3C**), the presented q-value was 2.44e-01, which did not pass our default significance threshold (q<0.2), indicating that adding the probiotic use to our model decreases the significance of the disease association. Furthermore, we noted that in model 3, probiotic use was significantly associated with *g:Lactobacillus* abundance, suggesting we investigate this transition further.

Indeed, many of the allergic infants received probiotics in their first year of life, thus the more significant association of *g:Lactobacillus* is with probiotics. To further investigate the contribution of probiotic use in this case, the user can stratify the association between disease and *g:Lactobacillus* by probiotic use, using the “facet by” option of the boxplot. Once the user selects probiotics as the faceted variable, the association between disease and *g:Lactobacillus* can be studied within the context of probiotics (with/without; **Figure 3D**). Once again, note that in the stratified view, the statistical analysis is using t-test in this specific context, rather than the MaAsLin2 systematic q-value (Methods).

## Discussion

A common goal of microbial community studies related to human epidemiology is to identify associations between microbial features and clinical variables. These studies must take into account additional factors, of clinical or environmental nature, that also impact the microbiome composition. Often, researchers turn to multivariate linear regression models to find the clinical associations while accounting for other measured confounding effects. Here, we present EasyMap, an interactive web-based tool that enables uploading custom input data, defining multiple such models, running the linear regression (using MaAsLin2 (Mallick et al., 2021)) and comparing the results across all tested models. Comparing the results allows for a better selection of model variables, without overfitting the data.

EasyMap can be run as an online webtool, and the full code is also available on github (Dahan, 2021), making the tool useful for researchers with varying levels of computational backgrounds. Currently, the web-based tool has a few hard-coded settings (such as the common data transformation; Methods), which are helpful in maintaining its ease of use, however user requests from github will be accommodated upon popular demand.

We developed EasyMap to assist us in analyzing data from our lab’s studies. It was built as a wrapper for MaAsLin2, with added visualization and comparison abilities, tailored for microbiome studies. However, it can be used in many additional contexts where multivariate linear models are commonly used, maintaining all its added value. EasyMap is also extremely useful for sharing results with collaborators, and enabling all participants to dig deeper in the analysis of their data.

## Data Availability Statement

The original contributions presented in the study are publicly available. These data and scripts can be found here: https://github.com/yassourlab/EasyMap. Further inquiries can be directed to the corresponding author.

## Ethics Statement

The studies involving human participants were reviewed and approved by Massachusetts General Hospital Institutional Review Board (IRB). Written informed consent to participate in this study was provided by the participants**’** legal guardian/next of kin.

## Author Contributions

ED developed the tool, VM evaluated the tool, MY guided the work, and ED and MY wrote the manuscript. All authors contributed to the article and approved the submitted version.

## Funding

Funding for this project is provided in part by the Azrieli Foundation and the Israeli Science Foundation.

## Conflict of Interest

The authors declare that the research was conducted in the absence of any commercial or financial relationships that could be construed as a potential conflict of interest.

## Publisher’s Note

All claims expressed in this article are solely those of the authors and do not necessarily represent those of their affiliated organizations, or those of the publisher, the editors and the reviewers. Any product that may be evaluated in this article, or claim that may be made by its manufacturer, is not guaranteed or endorsed by the publisher.
